# Exhalation of Rn-219 by patients treated with Radium-223

**DOI:** 10.1186/s40658-025-00719-6

**Published:** 2025-01-23

**Authors:** Carsten Wanke, Joerg Pinkert, Lilli Geworski, Bastian Szermerski

**Affiliations:** 1 Department for Radiation Protection and Medical Physics, Hannover Medical School, Carl-Neuberg- Str. 1, 30625 Hannover, Germany; 2 Bayer AG, Berlin, Germany

**Keywords:** Ra-223 dichloride, Exhalation, Rn-219

## Abstract

**Background:**

Treatment with Ra-223 dichloride is approved for the therapy of castration resistant prostate cancer (CRPC) with symptomatic bone metastases and no known visceral metastases in Europe since 2013, and Ra-223 is under discussion for labelling other molecules and nanoparticles. The direct progeny of Ra-223 is Rn-219, also known as actinon, a radioactive noble gas with a half-life of 3.98 s. This Rn-219 can be exhaled by patients while Ra-223 is present in the blood. Hence, direct measurements for assessing the exhalation of Rn-219 were performed for the first time in the context of the non-interventional multicenter study “RAPSODY”, a substudy to the international early access program, which aimed at assessing the radiation exposure of relatives of patients suffering from castration resistant prostate cancer with bone metastases and treated with Ra-223 dichloride in an outpatient setting, in order to investigate if this kind of method is functional and tolerated by the patients.

**Methods:**

Rn-219 was measured directly in patients’ exhalations using Alphaguard radon monitors (Saphymo, formerly Genitron, Frankfurt, Germany), originally intended for the measurement of Rn-222, and custom-made breath-test kits. Measurements were performed 20–30 min p. i. and 3–4 h p. i. In total, datasets from 21 administrations in 14 patients were obtained.

**Results:**

Although 75% of the measurement data 20–30 min p. i. and 35% of the measurement data 3–4 h p. i., respectively, were censored due to exceedance of the upper limit of the Alphaguards’ measurement range in the applied measurement setup, statistical data were derived based on the assumption of lognormal distributions. For measurements 3–4 h p. i., mean activity concentrations of Rn-219 in exhaled breath of approx. 4.4 kBq/l were obtained. In measurements 20–30 min p. i., the expectation value of the activity concentration of approx. 6 kBq/l, derived by statistical methods, was higher.

**Conclusions:**

Direct measurements using Alphaguard instruments are suitable for assessing the exhalation of Rn-219 by patients treated with Ra-223. The measurement method is well tolerated by the patients. Rn-219 is present in patients’ exhalations. Our results are in accordance with published data obtained using other, indirect measurement methods.

## Background

The treatment with Ra-223 dichloride is approved since 2013 in Europe [1] for the therapy of castration resistant prostate cancer (CRPC) with symptomatic bone metastases and no known visceral metastases. At present, this alpha emitter therapy is routinely performed, and Ra-223 is under discussion for labelling other molecules and nanoparticles [2–8]. The active agent contains the radionuclide Ra-223, an alpha emitter with a half-life of 11.43 d [9]. Most of the progeny of Ra-223 also emit alpha particles.

Direct progeny of Ra-223 is the noble gas radioisotope Rn-219, also known as actinon, with a half-life of only 3.98 s [9]. It decays via a series comprising three alpha and two beta decays to stable Pb-207.

It is well known that radon isotopes can be exhaled by humans that have taken up longer-lived mother nuclides. For example, Mayya et al. [10] measured the concentration of the isotope Rn-220 in exhaled breath of thorium plant workers, who are occupationally exposed to Th-232, Ra-228, Th-228 and Ra-224, all of which produce Rn-220 in their decay chain; and Kato and Ishikawa [11] used measurements of Rn-220 in the exhaled breath of patients, who received Thorotrast in the past, to assess the respective Thorotrast exposure. Therefore, it could be expected that patients receiving Ra-223 will exhale Rn-219.

The German Ministry for the Environment [12] and Stabin and Siegel [13] estimated that significant exposures due to the inhalation of Rn-219 are unlikely to occur. Petzold and Just [14] reported on measurements of patients’ exhalations with several radon measurement devices, indicating the necessity for further investigations. A statement on radiation protection measures when using Ra-223 dichloride in humans by the German Ministry for the Environment [12], in which a resulting dose for medical personnel after application is calculated, refers to measurements performed in Leipzig, Germany, without giving further references. Yamamoto et al. [15] reported elevated concentrations of Rn-219 and its progeny in the air of an examination room of a nuclear medicine facility when patients treated with Ra-223 were examined. Measurements of the Rn-219 concentration in the exhaled breath of patients are reported by Ooe et al. [16], who collected the exhaled air in plastic bags and applied gamma spectrometric measurements, thereby using an indirect method.

In this work we derive a method to obtain direct measurements of Rn-219 exhaled by patients. Generally, a direct method is desirable especially with regard to optimization purposes. For the measurements, the patients exhaled freely though a valve system into a reservoir, from which the exhaled air is pumped into the measurement device and analyzed with regard to the Rn-219 activity concentration. The measurements have been performed in the context of the study “RAPSODY - Measurement of radiation exposure of relatives and caregivers during outpatient therapy with Ra-223 dichloride in Germany”, which had been conducted as a substudy to the international early access program for Radium-223 dichloride (study 16216), a formal phase IIIb study [17], to evaluate the exposition of persons in the patient’s vicinity. Results of the measurements of the external radiation, of radioactivity in saliva and sweat and of contaminations in the patients’ homes and of an assessment of the exposure of relatives and caregivers are presented in [18].

## Methods

According to the study protocol, measurements of the Rn-219 exhaled by patients after administration of Ra-223 dichloride were to be performed 20–30 min p. i. and 3–4 h p. i. in the respective study center in the context of the RAPSODY study [18] using Alphaguard radon monitors (Saphymo, formerly Genitron, Frankfurt, Germany, own spelling: AlphaGUARD). The Alphaguard is a portable radon monitor originally intended for the measurement of Rn-222 and Rn-220, the latter isotope is also known as thoron. The Alphaguard turned out also to be suitable for the measurement of Rn-219 [19]. Two instruments were used for these measurements: an Alphaguard P30F, after the first patient measurements upgraded to PQ2000F (see below), and an Alphaguard PQ2000pro. Both instruments were operated in the flow mode with a measurement cycle of one minute, which means that the gas to be measured is continuously pumped through the measurement chamber of the instrument and the signal is automatically averaged and displayed over measurement times of one minute. The measurement ranges for Rn-222 of the instruments used in this work are stated to be 2 Bq/m^3^–3·10^4^ Bq/m^3^ for the model P30F and 2 Bq/m^3^–2·10^6^ Bq/m^3^ for the models PQ2000F and PQ2000pro.

Details on the measurement principle are given in the user manual [20] and by Genrich [21]. The measurement principle of the instrument is that of an ionization chamber. For readings greater than 600 kBq/m^3^, the signal is evaluated by the DC integrating current measurement technique, making the instrument suitable also for the measurement of Rn-219, which has been demonstrated in [19]. For readings less than 600 kBq/m^3^, further methods of signal evaluation are applied by the instruments and contribute to the instrument reading. The behavior of the instrument and the response to Rn-219 in the range below readings of 600 kBq/m^3^ have not been investigated in frame of [19], hence instrument readings below 600 kBq/m^3^ could not be evaluated in this work.

In [19], we derived a conversion factor from instrument reading to Rn-219 concentration of *a*_Rn−219_ = 2.3 *R*, where *a*_Rn−219_ is the concentration of Rn-219 in air and *R* is the instrument reading. The conversion factor was obtained using Rn-219 sources and the same measurement setup as used in this work for patient measurements. As a filter (see below) retains decay products of Rn-219 possibly exhaled by the patients, it can be assumed that only Rn-219 exhaled by the patient enters the measurement chamber and contributes to the measurement signal. Therefore, the factor derived in [19] is deemed appropriate and is used as a conservative estimate for the conversion of instrument readings to Rn-219 concentration for readings between 600 kBq/m^3^ and 2000 kBq/m^3^ in this work.

For improved readability, concentrations are given in this work in units of kBq/l. The instruments display higher concentrations as “>2000 kBq/m^3^”, which convert to Rn-219 concentrations of > 4.6 kBq/l in the measurement chamber, however no precise numerical value can be stated. An instrument reading of 600 kBq/m^3^ converts to a Rn-219 concentration in the measurement chamber of the Alphaguard of 1.38 kBq/l; measurement results below this value are evaluated as < 1.38 kBq/l in frame of this work.

Two pumps (both Saphymo, formerly Genitron, Frankfurt, Germany) were used, an Alpha Pump (own spelling: AlphaPUMP) with a maximum flow rate of 1 l/min and a Lab Pump (own spelling: LabPUMP) with a maximum flow rate of 4 l/min. Both pumps were operated with the maximum flow rates for patient measurements.

**Fig. 1:**
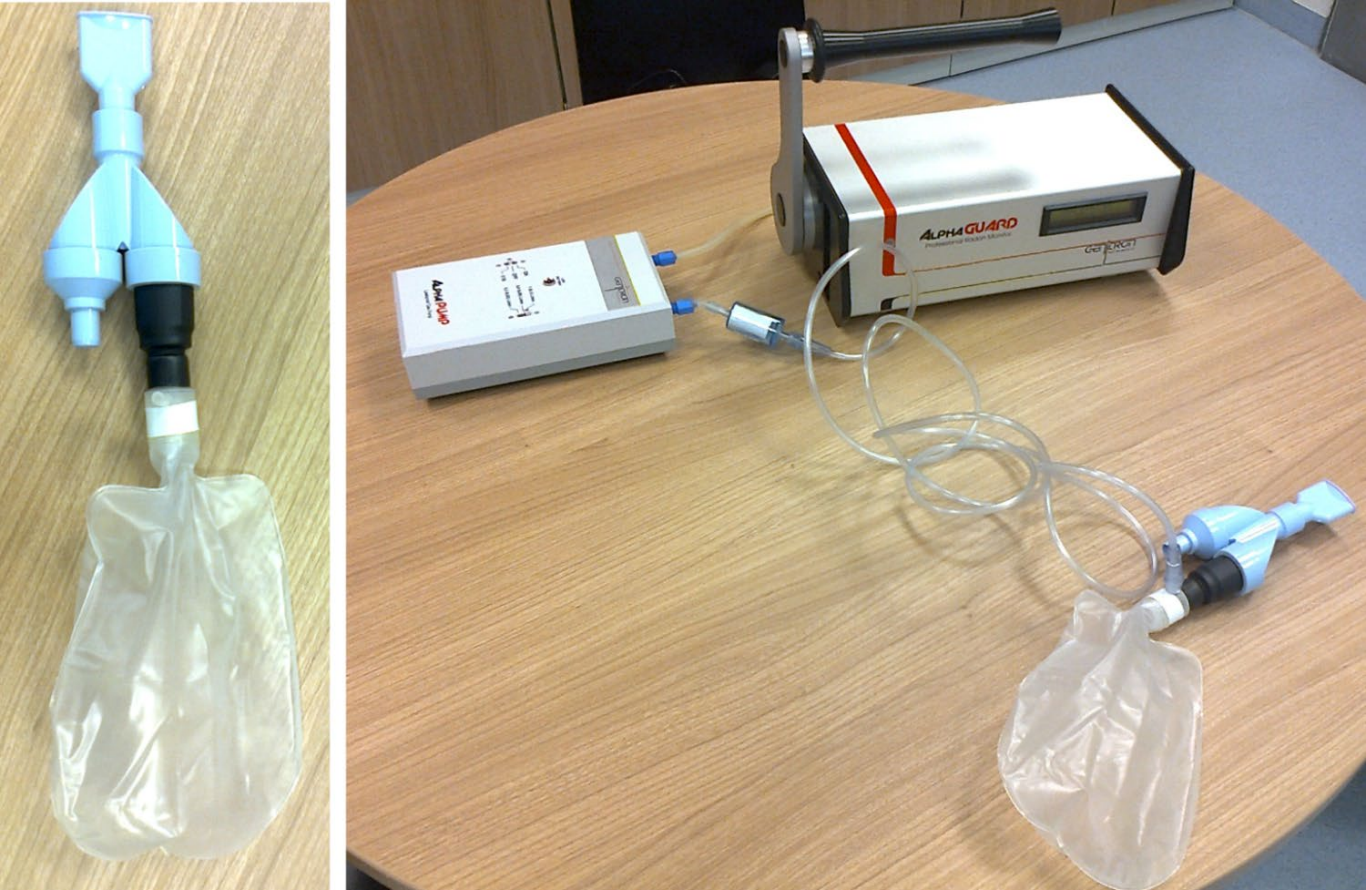
Left: breath test kit, Right: measurement setup

The study centers were supplied with the Alphaguard instruments, detailed instructions for the measurements and custom-made breath test kits for sampling patients’ exhalations. The test kits (see Fig. 1) were made from Technegas™ patient administration sets (Cyclomedica Germany), which are originally intended for the application of carbon-based nanoparticles labelled with Tc-99 m for lung ventilation SPECT imaging. The administration sets are equipped with a valve system and were combined with reservoirs from oxygen masks, see Fig. 1. Using such a test kit, the patient can in- and exhale freely through the mouth. The exhaled breath is directed into the reservoir and can be sampled via the oxygen connector of the reservoir bag. For the measurement of the Rn-219 concentration in the exhaled air, the tube delivered with the oxygen masks was connected to the reservoir and to the pump feeding the Alphaguard, see Fig. 1. Between tube and pump, a filter (visible in Fig. 1 before the intake of the pump) was inserted to prevent particles, progeny of Rn-219 and moisture from entering the pump.

For sampling, the patient was asked to take the mouthpiece, seal it with his lips and breathe normally through the mouth for a few minutes. Two instrument readings were recorded manually at a time interval of 3–5 min after the measurement signal became stable. The background value was recorded before the patient entered the room; as the background values are smaller than the measurement readings by several orders of magnitude, the background was negligible. The instrument readings were converted to Rn-219 concentrations applying both the conversion factor from instrument reading to Rn-219 concentration stated above as well as the correction for decay in the tube: The volume of the tube can be estimated as approximately 20 ml. From the flow rates and the tube volume, travelling times of the gas through the tube can be estimated to approx. 1.2 s and 0.3 s for pump rates of 1 l/min and 4 l/min, respectively. Taking the half-life of Rn-219 into account, this leads to a decay of Rn-219 of 23.4% (pump rate 1 l/min) and 5.4% (4 l/min). The measurement results were corrected for this decay in the tube.

The two recorded readings were to be combined to yield one single data point per patient and time point. As it turned out that a number of results was censored (see results), the following approach was chosen: When both single readings recorded were uncensored (point) values, the final measurement result was obtained as arithmetic mean of the two single values and stated as point value. When both recordings indicated that the measurement range of the instrument was exceeded with identical readings, the final result is stated as censored “greater than” value. When one of the recordings was an uncensored value and the other indicated an exceedance of the measurement range, the final result is given as “greater than the lower value”.

From these results, the geometric mean and geometric standard deviation as well as the 95th percentile were derived as statistical measures assuming lognormally distributed data. As a large number of data points were censored due to exceedance of the upper limit of the Alphaguards’ measurement range, it seems likely that statistical parameters derived from uncensored point values, neglecting the censored data, underestimate the true statistical measures. Therefore, we chose the following approach for deriving statistical parameters: q-q-plots of the data were constructed using Microsoft Excel. In the q-q-plots, an exponential dependence of the results on the theoretical quantiles of a lognormal distribution, which converts to a linear dependence of the natural logarithm of the results on the theoretical quantiles of a normal distribution, was assumed. Data censored due to exceedance of the upper limit of the measurement range were excluded. From the dependences derived this way, imputations for the “greater than” data points as well as statistical measures, e.g. means, standard deviations and percentiles, were be calculated using the dependences derived from the measured “real” data.

## Results

In the context of the RAPSODY study, datasets from 21 administrations of Ra-223 in 14 patients were obtained in total with this novel, direct measurement method, with a maximum of two datasets per patient. The measurements were well tolerated by the patients. Rn-219 was clearly detectable in the patients’ exhalations. As mentioned above, one of the two instruments was an Alphaguard P30F with an upper measurement limit of 30 kBq/m^3^. After the first measurements with this instrument it turned out that the measurement range is not sufficient for measurements of exhaled Rn-219 performed as described above, thus an upgrade of this instrument to the PQ2000F version was necessary. Because of this, four data sets obtained with the Alphaguard P30F were discarded. Measurement data of the exhalation of Rn-219 at both time points (20–30 min p. i. and 3–4 h p. i.) that could be evaluated were recorded after 16 administrations. In one further administration only the later measurement was performed.

Measurements with the PQ2000 instruments displayed readings indicating concentrations exceeding the upper measurement limit of the instrument in a number of cases. As mentioned above, the upper limit of the measurement range corresponds to a Rn-219 concentration of 4.6 kBq/l in the measurement chamber of the instrument. Taking the flow rates of the pumps into account, this upper limit corresponds to exhaled concentrations of Rn-219 of > 4.8 kBq/l and > 5.7 kBq/l, respectively, for flow rates of 1 l/min and 4 l/min. In two datasets, one of the recordings was an uncensored value and the other indicated an exceedance of the measurement range. In these cases, the final result is given as “greater than the lower value”. In one dataset, the first recording was an uncensored value and the second recording showed a significantly lower reading below 600 kBq/m^3^, where no reliable conversion of instrument reading to Rn-219 concentration was available. This second recording was interpreted to be an outlier due to an incorrect use of the sampling kit leading to an aspiration of fresh air, hence only the first recording was taken into evaluation as final value. In one further dataset, both readings were below 600 kBq/m^3^, which convert to less than 1.7 kBq/l. The final result of this dataset is given as < 1.7 kBq/l.

In the measurements performed 20–30 min p. i., the final results of 12 out of 16 measurements (75%) exceeded the upper limit of the measurement range. In measurements performed 3–4 h p. i., ten out of 17 measurements yielded “real” final results, while the final results of six measurements exceeded the upper limit of the measurement range, and in one further measurement the final result was < 1.7 kBq/l. The results are presented in Table 1.

**Table 1:** Results

Number	Applied Activity in MBq	Decay factor	Conversion factor	20–30 min *p*. i.				3–4 h *p*. i.			
				Measurement result 1 in kBq/l	Measurement result 2 in kBq/l	Final result in kBq/l	Final result per unit activity applied in kBq/(l MBq)	Measurement result 1 in kBq/l	Measurement result 2 in kBq/l	Final result in kBq/l	Final result per unit activity applied in kBq/(l MBq)
1	4.37	1.054	2.3	> 4.61	> 4.61	> 4.61	> 1.05	> 4.82	> 4.82	> 4.82	> 1.10
2	4.36	1.054	2.3	4.61	4.61	4.61	1.06	4.61	4.82	4.82	1.11
3	4.87	1.054	2.3	4.82	4.82	4.82	0.99	4.82	4.82	4.82	0.99
4	3.10	1.054	2.3	> 4.85	> 4.85	> 4.85	> 1.57	3.87	< 1.45^1^	3.87	1.25
5	5.06	1.054	2.3	> 4.85	> 4.85	> 4.85	> 0.96	> 4.85	4.29	> 4.29	> 0.85
6	3.33	1.234	2.3	5.37	5.37	5.37	1.61	1.96	2.12	2.04	0.61
7	3.99	1.234	2.3	> 5.68	> 5.68	> 5.68	> 1.42	4.63	4.43	4.53	1.13
8	3.75	1.054	2.3	> 4.85	> 4.85	> 4.85	> 1.29	> 4.85	> 4.85	> 4.85	> 1.29
9	4.62	1.234	2.3	> 5.68	> 5.68	> 5.68	> 1.23	5.53	> 5.68	> 5.53	> 1.20
10	4.43	1.234	2.3	> 5.68	> 5.68	> 5.68	> 1.28	> 5.68	> 5.68	> 5.68	> 1.28
11	5.49	1.234	2.3	> 5.68	> 5.68	> 5.68	> 1.03	4.42	3.66	4.04	0.74
12	5.12	1.234	2.3	> 5.68	> 5.68	> 5.68	> 1.11	< 1.70	< 1.70	< 1.70	< 0.33
13	4.89	1.234	2.3	> 5.68	> 5.68	> 5.68	> 1.16	3.28	3.04	3.16	0.65
14	5.20	1.234	2.3	> 5.68	> 5.68	> 5.68	> 1.09	3.32	2.98	3.15	0.61
15	3.77	1.054	2.3	4.85	4.18	4.51	1.20	3.30	3.30	3.30	0.87
16	3.74	1.054	2.3	> 4.85	> 4.85	> 4.85	> 1.30	> 4.85	> 4.85	> 4.85	> 1.30
17	4.33	1.054	2.3	-	-	-	-	2.58	2.39	2.48	0.57

^1^ Not taken into evaluation, cf. Section 3

**Fig. 2:**
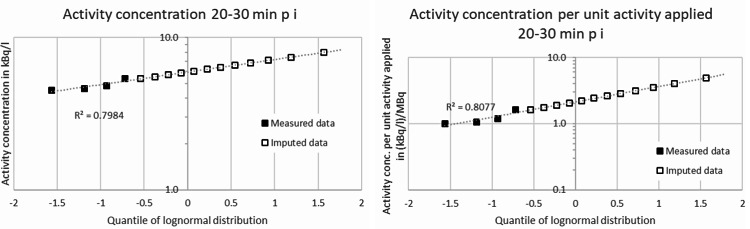
q-q-plots for activity concentrations (left) and activity concentrations per unit activity applied (right) from measurements 20–30 min p. i.

**Fig. 3:**
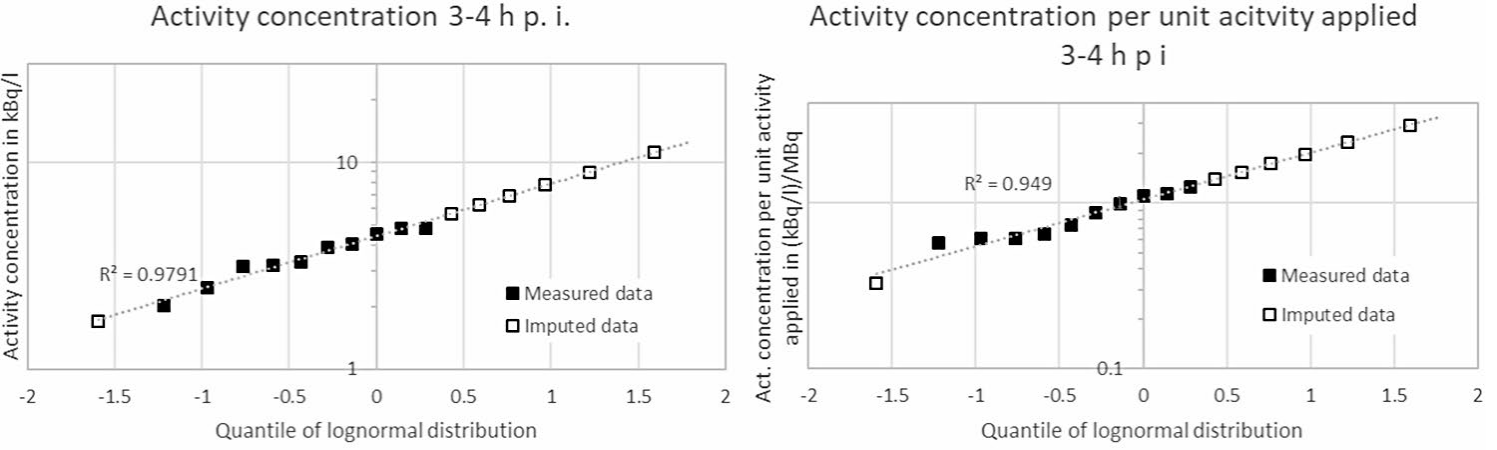
q-q-plots for activity concentrations (left) and activity concentrations per unit activity applied (right) from measurements 3–4 h p. i.

From the uncensored data and taking a conservative imputation of 1.7 kBq/l for the result of < 1.7 kBq/l in the measurements 3–4 h p. i. into account, ranges of the activity concentration of Rn-219 in exhaled breath of 4.51–5.37 kBq/l (median 4.71 kBq/l) for the time point 20–30 min p. i. and 2.04–4.82 kBq/l (median 3.58 kBq/l) for the time point 3–4 h p. i. would be obtained. From the dependences derived this way, imputations for the “greater than” data points as well as statistical measures, e.g. means, standard deviations and percentiles, can be calculated using the dependences derived from the measured “real” data. The q-q-plots for the results from measurements, given as measured activity concentrations and as activity concentrations per unit applied activity, each 20–30 min p. i. and 3–4 h p. i., are shown in Figs. 2 and 3, respectively. In view of the *R*^2^ values shown in Figs. 2 and 3 it can be concluded that the data can be described sufficiently well by a lognormal distribution. Statistical data are given in Table 2.

**Table 2:** Statistical data derived from the measurement results

Time point	20–30 min p. i.		3–4 h p. i.	
Number of analyzable datasets	16		17	
- Of which measured real data	4		11*	
- Of which censored data	12 (75%)		6 (35%)	
Quantity	Activity concentration	Activity concentration per unit applied activity	Activity concentration	Activity concentration per unit applied activity
Assumed distribution	lognormal	lognormal	lognormal	lognormal
*R* ^2^	0.80	0.81	0.98	0.95
Geometric mean and standard deviation	(5.94∙1.18^± 1^) kBq/l	(2.14∙1.60^± 1^) kBq/(l MBq)	(4.40∙1.67^± 1^) kBq/l	(1.05∙1.78^± 1^) kBq/(l MBq)
95th percentile	8.11 kBq/l	5.15 kBq/(l MBq)	11.52 kBq/l	3.05 kBq/(l MBq)

* including 1 “less than” value

## Discussion

This work presents first results of novel, direct measurements of Rn-219 exhaled by patients treated with Ra-223 dichloride. We obtained geometric means and standard deviations of the activity concentrations of Rn-219 in exhaled breath measured 20–30 min p. i. and 3–4 h p. i. of approx. (5.94∙1.18^± 1^) kBq/l and (4.40∙1.67^± 1^) kBq/l, respectively, assuming lognormal distributions of the data. Regarding the activity concentrations per unit administered activity, we obtained geometric means and standard deviations of (2.14∙1.60^± 1^) kBq/l and (1.05∙1.78^± 1^) kBq/l measured 20–30 min p. i. and 3–4 h p. i., respectively, again under the assumption of lognormally distributed data. The assumption of lognormal distributions appears generally to be reasonable when biological processes are involved [22]. The calculated *R*^2^-values for the data 3–4 h p. i. of 0.93–0.98 (cf. Figure 3) indicate that at least the data measured 3–4 h p. i. might be adequately described by a lognormal distribution. As the “real” data measured 20–30 min p. i. are too sparse, no statement on the adequacy of a lognormal distribution of these data can be made. The assumption of normally distributed data, however, yields arithmetic means for both time points that are approximately equal to the geometric means stated above.

Measurements of Rn-219 exhaled by patients after administration of Ra-223 dichloride were described by only a few publications so far. Petzold and Just reported measurements with several radon measurement devices [14], including measurements with an Alphaguard which might be similar to those presented in this work, and claim to have measured a mean concentration of approx. 25 kBq/l in the exhaled breath of one patient “in the first minutes after administration”. However, the article did not give further details on the measurements, the calibration or the patient population, but indicated the necessity for further investigations. Yamamoto et al. [15] reported elevated concentrations of Rn-219 and its progeny in an examination room of a nuclear medicine facility when patients treated with Ra-223 were examined. A publication by Ooe et al. [16] presents the results of determinations of the Rn-219 concentration in the exhaled breath of patients. Ooe et al. collected the exhaled air in plastic bags. After decay of Rn-219 the air was removed from the bags through a glass fibre filter, the bags and filters were then measured using gamma spectrometry with a germanium detector for the quantification of the daughter nuclide Bi-211. The average activity concentrations in the exhaled air measured 1 min and 5 min after administration of Ra-223 dichloride is stated to be (90 ± 56) Bq/ml and (28 ± 9) Bq/ml, respectively, which convert to (90 ± 56) kBq/l and (28 ± 9) kBq/l, respectively, in measurement units used in this work. In contrast to our direct measurements, Ooe et al. used an indirect method for assessing the Rn-219 concentration in exhaled air.

The activity concentration of Rn-219 in exhaled breath can be assumed to follow the same dynamics as the concentration of Ra-223 in blood, as no significant accumulation of Rn-219 generated from Ra-223 in other organs or tissues can occur in blood due to the short half-life of 3.98 s. Hence, for comparison of measurement data taken at different time points, a conversion should be possible using pharmacokinetics data of Ra-223 concentration in blood. Nilsson et al. [23] and Yoshida et al. [24] have presented diagrams on pharmacokinetic data on the Ra-223 concentration in blood after administration. From both diagrams it can be estimated that the activity concentration of Ra-223 in blood 3–4 h p. i. should be approx. 3 times lower than the activity concentration in blood 20–30 min p. i. Data on the blood clearance of Ra-223 supporting a factor of approx. 3 are also presented in [25]. The arithmetic and geometric means of the activity concentrations per unit activity administered measured in this work at 3–4 h p. i. are lower than those measured at 20–30 min p. i. by a factor of approx. 2. In view of the uncertainties and variations generally present in experimental exhalation measurements as well as in the pharmacokinetic data and models, both seem in agreement. On the other hand, Carrasquillo et al. [26] published pharmacokinetic parameters from which a theoretical factor as large as 4.1–5.5 between blood concentrations 20–30 min p. i. and 3–4 h p. i. can be calculated. This could indicate that the statistical data derived for the measurements 20–30 min p. i. underestimate the true values, possibly due to the large proportion of censored data.

The blood clearance data in [25] suggest a factor of about 22.5 between the activity concentrations in blood measured 1 min p. i. and 4 h p. i. Using the mean activity concentration of Rn-219 in exhaled breath measured 3–4 h of 4.4 kBq/l and applying the factor of 22.5, an estimated concentration for 1 min p. i. of 99 kBq/l can be calculated. This value agrees perfectly well with the data of Ooe et al. [16].

Main limitation of this study is the large number of censored data points. This is due to the fact that the exhaled Rn-219 concentrations in combination with the flow rates and tube lengths applied lead to radon concentrations in the measurement chambers of the Alphaguard instruments that are in the order of or exceed the upper limit of the measurement range. As a consequence, the statistical data presented in Table 2, which are derived from assumed distributions, might not aptly describe the real distributions, as extreme values would be undetectable. On the other hand, the majority of data obtained in the measurements 3–4 h p. i. are not censored, and calculating activity concentrations for other time points after administration based on published data on pharmacokinetics yields results that agree with data from other publications very well. Hence, it can be assumed that the data presented in Table 2 describe the real distributions reasonably well. If measurements as described in this publication are repeated in the context of another study, it should be considered to use greater tube lengths or lower pump rates. Both lead to longer travelling times allowing the Rn-219 to physically decay further and eventually leading to lower concentrations in the measurement chamber, cf [19]. Hence, the resulting instrument readings are more likely to be evaluable. The results could then be used to verify the statistical measures obtained in this work and the assumptions on the distributions.

## Conclusions

Ra-223 decays into Rn-219, a noble gas, and exhalation of Rn-219 is to be expected in patients treated with radiopharmaceuticals containing the radioisotope Ra-223. In this work, direct measurements of Rn-219 in patients’ breath using Alphaguard radon monitors originally intended for the measurement of the Isotope Rn-222 and custom-made breath-test kits allowing free breathing were applied to assess the exhalation. Measurements were performed 20–30 min p. i. and 3–4 h p. i.

It could clearly be shown that Rn-219 is present in patients’ exhalations. Although a number of data was censored due to exceedance of the upper limit of the Alphaguards’ measurement range, statistical data were derived based on the assumption of a normal distribution and a lognormal distribution, respectively. For measurements 3–4 h p. i., mean activity concentrations of Rn-219 in exhaled breath of approx. 4.4 kBq/l were obtained. Calculating activity concentrations for other time points after administration from this value based on published data on pharmacokinetics yields results that agree with data from other publications very well.

## Data Availability

All data analysed during this study are included in this published article.
